# *In situ* hydrogen loading on zirconium powder

**DOI:** 10.1107/S1600577515009054

**Published:** 2015-06-26

**Authors:** Tuerdi Maimaitiyili, Jakob Blomqvist, Axel Steuwer, Christina Bjerkén, Olivier Zanellato, Matthew S. Blackmur, Jérôme Andrieux, Fabienne Ribeiro

**Affiliations:** a Materials Science and Applied Mathematics, Malmö University, Östra Varvsgatan 11 A, Malmö, Skane 20506, Sweden; b Max IV Laboratory, Lund University, Ole Römers väg, Lund, Skane 22100, Sweden; c Nelson Mandela Metropolitan University, Gardham Avenue, Port Elizabeth 6031, South Africa; d PIMM, Ensam - Cnam - CNRS, 151 Boulevard de l’Hôpital, Paris 75013, France; e Materials Performance Centre, School of Materials, The University of Manchester, Manchester M1 7HS, UK; f ID15-B, European Synchrotron Radiation Facility, 6 rue J Horowitz, Grenoble 38043, France; g Laboratoire des Multimatériaux et Interfaces, Université de Lyon, 43 Bd du 11 novembre 1918, Lyon 69100, France; h Institut de Radioprotection et Sûreté Nucléaire, IRSN, BP 3, 13115 Saint-Paul Lez Durance, France

**Keywords:** zirconium hydride, synchrotron X-ray diffraction, *in situ* hydrogen charging, hydrogen-induced degradation, phase transformation

## Abstract

For the first time, various hydride phases in a zirconium–hydrogen system have been prepared in a high-energy synchrotron X-ray radiation beamline and their transformation behaviour has been studied *in situ*. First, the formation and dissolution of hydrides in commercially pure zirconium powder were monitored in real time during hydrogenation and dehydrogenation, then whole pattern crystal structure analysis such as Rietveld and Pawley refinements were performed. All commonly reported low-pressure phases presented in the Zr–H phase diagram are obtained from a single experimental arrangement.

## Abstract 2

Commercial-grade Zr powder loaded with hydrogen *in situ* and phase transformations between various Zr and ZrH_*x*_ phases have been monitored in real time.

## 1. Introduction   

Mechanical properties such as high strength, high melting point and excellent corrosion resistance in almost all environments make zirconium (Zr), and its alloys, widely used as engineering materials in many industries ranging from everyday consumer goods to the chemical industry. The most important application of Zr is in the nuclear power industry because of its neutron transparency (Zuzek *et al.*, 1990 ▸; Steuwer *et al.*, 2009 ▸; Olsson *et al.*, 2014 ▸; Lanzani & Ruch, 2004 ▸). In nuclear power reactors, Zr alloys are mostly used as fuel rod cladding to hold the nuclear fuel pellets inside the reactor core. During reactor operation the environment inside the reactor core becomes harsh (250–350°C, 7–15 MPa) and Zr alloys undergo corrosion, which also produces free hydrogen. Some of the hydrogen released through corrosion is absorbed into the Zr cladding material and eventually leads to the precipitation of hydrides once the solubility limit of hydrogen in Zr is exceeded (Singh *et al.*, 2005 ▸). As the hydride phases have a larger unit volume than the solid solution phase (α-Zr), the formation of hydrides will introduce internal stresses, which may lead to delayed hydride cracking (Coleman & Hardie, 1966 ▸; Northwood & Kosasih, 1983 ▸; IAEA, 2010 ▸). Thus, the formation of hydrides is a potential issue during extended fuel burn-up and when reactors are taken off-line and cooled to ambient temperature. To extend the life span of claddings and avoid catastrophic failure, it is necessary to identify the nature of various Zr hydride phases and their exact structures.

**Figure 1:**
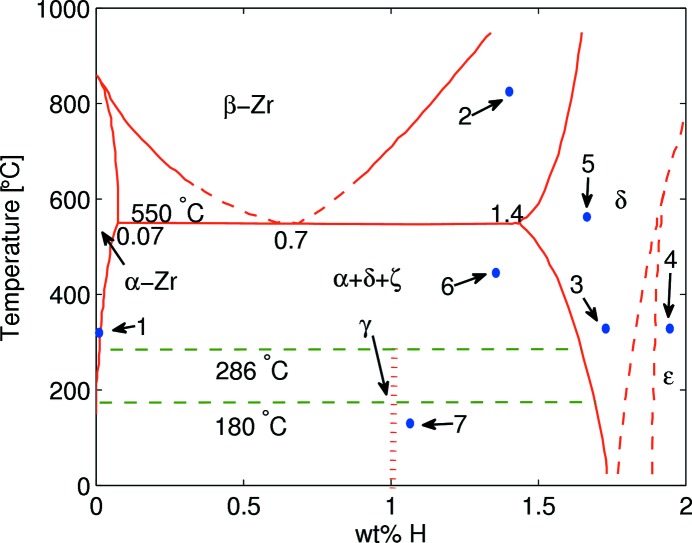
Zr–H phase diagram (Zuzek *et al.*, 1990 ▸) with approximate positions of observed phases. The point identification number is the same as in Fig. 3 ▸.

As shown in the zirconium–hydrogen (Zr–H) binary phase diagram (Fig. 1 ▸), at ambient pressure and low temperature (≤868°C) Zr exists as a hexagonal-close-packed (h.c.p.) structure with the Mg structure type (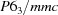, *a* = 3.2316 Å, *c* = 5.1475 Å), which is commonly referred to as the α-phase. When the temperature exceeds 868°C, the α-phase transforms into a body-centred-cubic (b.c.c.) structure known as β-Zr phase, which is of the W structure type (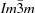, *a* = 3.6090 Å). With respect to hydrides, it is evident that, depending on hydrogen concentration and quenching rate, Zr hydride can exist in two stable and two metastable phase forms. The two stable hydride phases are: face-centred-cubic (f.c.c.) ZrH_1.66_, known as the δ-Zr hydride phase which is of the CaF_2_ structure type (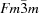, *a* = 4.7783 Å), and face-centred tetragonal (f.c.t.) ZrH_2_, known as the ∊-Zr hydride phase with the ThH_2_ structure (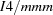, *a* = 4.9689 Å, *c* = 4.4479 Å). Two metastable hydride phases include the metastable f.c.t. γ-hydride phase with structure type ZrH (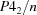, *a* = 4.592 Å, *c* = 4.970 Å), and one newly observed trigonal ζ-hydride (*P*3*m*1, *a* = 3.242 Å, *c* = 10.33292 Å) (Zuzek *et al.*, 1990 ▸; Steuwer *et al.*, 2009 ▸; Olsson *et al.*, 2014 ▸; Lanzani & Ruch, 2004 ▸; Okamoto, 2006 ▸; Zhao *et al.*, 2008 ▸). Basic crystallographic data of all these reported phases are tabulated in Table 1 ▸.

Although the existence of various Zr hydrides and related problems have been known for more than 50 years, there are still many discrepancies regarding the stability, crystal structure, formation mechanisms and the transformation temperature of γ-ZrH (Zuzek *et al.*, 1990 ▸; Steuwer *et al.*, 2009 ▸; Lanzani & Ruch, 2004 ▸; Kerr, 2009 ▸; Tulk *et al.*, 2012 ▸; Root *et al.*, 2003 ▸; Kolesnikov *et al.*, 1994 ▸). It is also not clear at which precise hydrogen concentration (Bowman *et al.*, 1983 ▸; Cassidy & Wayman, 1980 ▸; Veal *et al.*, 1979 ▸), at which temperature or in which kind of transformation order (Cassidy & Wayman, 1980 ▸; Mueller *et al.*, 1968 ▸; Ivashchenko *et al.*, 2009 ▸; Cantrell *et al.*, 1984 ▸; Barraclough & Beevers, 1970 ▸; Moore, 1969 ▸) the f.c.t. 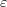-ZrH_*x*_ transforms into f.c.c. δ-ZrH_*x*_. Such discrepancies arise because of the high diffusivity of hydrogen at low temperature, structural similarities between various phases, the extended hydrogen concentration interval of hydride phases and the strong influence of other impurity elements in the Zr–H system. From the literature, it is known that the stability of various Zr hydride phases depends on alloying elements and internal stresses (Steuwer *et al.*, 2009 ▸; Lanzani & Ruch, 2004 ▸). In order to capture the kinetics of hydrogen absorption and desorption, to record fast phase transformation and perform phase-structural characterization with high accuracy in Zr–H systems, it is important to perform *in situ* hydrogen loading studies. However, to the best of our knowledge there have been no *in situ* hydrogen charging studies performed on high-purity Zr powder at a high-energy synchrotron radiation facility. In addition, in many of these reported studies the structures are based on experiments carried out on *ex situ* hydrided polycrystal alloy samples using different techniques and facilities. Thus, there might be alloying/impurity elements effects or experimental discrepancies involved.

In this work, we have performed *in situ* hydrogen loading experiments at the beamline ID15B at the European Synchrotron Radiation Facility (ESRF) in Grenoble, France. An on-site high-pressure/high-temperature capillary system (Andrieux *et al.*, 2014 ▸) was used to hydrogenate high-purity Zr powder. This produced the commonly reported five phases (excluding the ζ-phase) in the Zr–H system in one single setup, and their formation and transformation behaviour could be monitored in real time. Rietveld analyses (Coelho, 2004 ▸) were performed to determine the crystal structure of the various phases. In this paper we will not address any structure- or transformation-related questions. Instead the focus of this report is on the hydride preparation route and setups.

## 2. Experimental setup and data processing   

### 2.1. Sample for *in situ* measurements   

**Figure 2:**
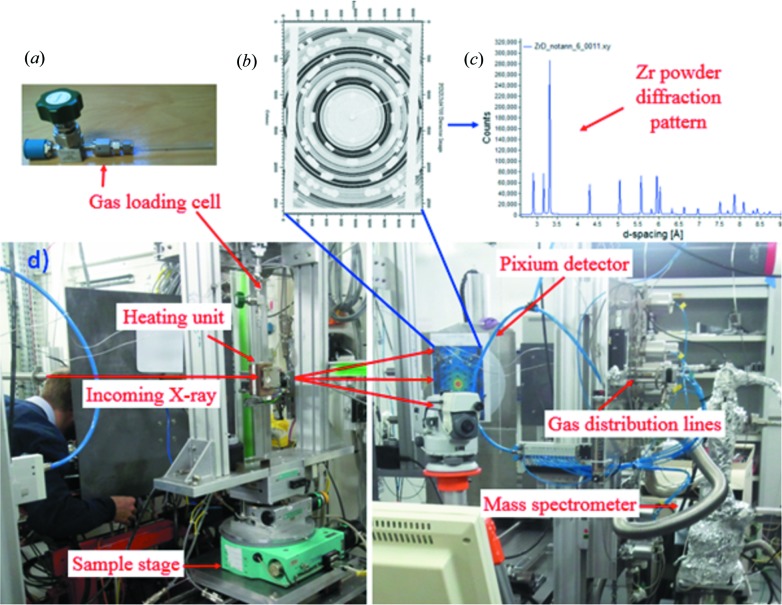
The hydrogenation and beamline setup, with (*a*) gas loading cell, (*b*) raw image, (*c*) resulting diffractogram after correction and integration, and (*d*) the arrangement of the actual setup.

The powder sample that was used in the experiments was prepared very carefully. First, a commercial-grade pure zirconium powder (99.2% purity) with maximum particle size *ca* 45 µm was purchased from Goodfellow Ltd, Huntingdon, England (ZR006015). The nominal composition of the sample is shown in Table 2 ▸. In order to dissolve any hydrides that may have formed earlier or during preparation, the sealed cells containing Zr powder were first baked at ∼700°C for ∼7.5 h, and then at ∼1000°C for 5 h until an ultra-high-vacuum level was achieved. Then, the Zr powder was filled into a single-ended closed sapphire single-crystal container (Fig. 2 ▸ *a*) in an argon environment to prevent contamination or oxidation.

### 2.2. Beamline setup   

The experiment was performed at ID15B at ESRF. ID15B is one of the side-stations of the powerful asymmetric multipole wiggler (the insertion straight section holds a seven-pole 1.84 T wiggler) beamline ID15. As a side-station, the ID15B cannot receive a white X-ray beam, instead it receives a monochromatic beam from a horizontally focusing monochromator located in the optics hutch. A fixed high-energy monochromatic X-ray beam of 87.7 keV was generated by a Si(511) bent Laue monochromator. The beam size was defined by a set of tungsten slits located just prior to the sample stage. A large two-dimensional flat-panel detector (Pixium Trixell 4700, Thales) positioned far from the specimen (∼1.2 m) was used for data collection (Andrieux *et al.*, 2014 ▸; Daniels, 2008 ▸). The high-pressure and high-temperature gas loading system together with a custom-made mass spectrometer available at ID15B (Andrieux *et al.*, 2014 ▸) were used for hydrogen reaction and detection. The complete hydrogen charging setup is shown in Fig. 2 ▸. In order to avoid a diffraction contribution from the single-crystal sapphire container that was used for powder holding, a lead mask was placed in front of the detector, and these masked areas can be seen as white spots on diffraction rings (Fig. 2 ▸ *b*).

### 2.3. Hydrogen charging and data collection   

For data acquisition, 0.2 s (hydrogenation) and 20 s (dehydrogenation) of exposure time were used based on observed phase transformation rates. The wavelength of the X-ray beam was calibrated using a standard LaB_6_ specimen (SRM 660a, *a* = 4.1569162±0.0000097 Å) to λ = 0.142352 Å. During the whole measurement, the wavelength was kept constant.

As the inner diameter of the capillaries that were used for holding samples was ∼3 mm, we selected a 0.3 mm × 0.3 mm beam size to ensure good diffraction signals from the specimens. For the purpose of slowing down the hydrogen diffusion rate and to be able to record all possible phase transitions, we controlled the loading pressure to between 0.05 and 1 MPa depending on the observed phase transformation rate. Considering the length and focus of this paper, only data collected from a measurement performed under static applied pressure (0.05 MPa) is presented here. The sample temperature was constantly measured with a thermocouple that was connected to the end of the capillary, as shown in Fig. 2 ▸(*a*).

**Figure 3:**
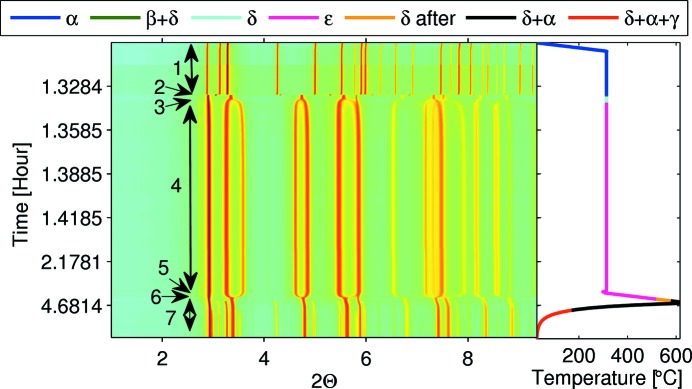
Complete phase transformation during hydrogenation and dehydrogenation with different heating cycles. The colour-coded legends at the top correspond to a part of the thermal history curve coloured the same way.

In order to establish the stability and formation mechanisms of the hydride phases, the measurement started at room temperature with pure powder, and data were collected continuously at various times as a function of temperature. The heating and cooling rates were controlled to ∼10°C min^−1^. The detailed thermal history and corresponding diffraction map are shown in Fig. 3 ▸. As shown, the hydrogen gas was introduced once the system temperature reached 300°C, then the system was kept fixed for 3.5 h until no further phase transformation was observed. At the end, the system was heated up to 613°C, and then cooled back to room temperature at the same rate. All diffraction patterns collected from the measurements are shown as an assembled plot in Fig. 3 ▸.

### 2.4. Data processing   

Raw data were first corrected for two-dimensional flat-panel artefacts and polarization of the synchrotron X-ray beam as described by Andrieux *et al.* (2014 ▸). The normalized and corrected data were first integrated [using *MATLAB* (The MathWorks Inc, version 7.10.0)] and then analysed using the crystal structure analysis software packages *Topas-Academic* (Coelho, 2004 ▸) and *GSAS* (Larson & Von Dreele, 2000 ▸).

As the diffraction pattern is a convolution of instrument, sample and background contributions, we first used the Pawley method on the diffraction pattern of a standard LaB_6_ sample collected with the same setup prior to measurement to determine the instrument/profile function and to calibrate the wavelength of the X-ray beam. For instrumental function, the modified Thompson–Cox–Hastings pseudo-Voigt function (Coelho, 2004 ▸) was used together with a simple axial model. After calibration, refinement of the Zr powder diffraction pattern was carried out according to the refinement parameter turn-on sequence suggested by Young (1995 ▸; Table 1.5).

Preliminary crystal structure information for various Zr and Zr hydride phases, which were needed for whole pattern structure analysis using the Rietveld and Pawley method, were obtained from the literature (Zuzek *et al.*, 1990 ▸). All structural data used, such as reference and refined parameters, are tabulated in Table 1 ▸. Both Pawley and Rietveld refinements were performed on the full diffraction peak range which spans from 0.37 to 9.46° in 2θ and 21.71 to 0.86 Å in *d*-spacing. The background was fitted with a Chebyshev function (Coelho, 2004 ▸) with five coefficients and the zero-shift error calibrated from LaB_6_ was kept fixed. As the material in question is a fine-grained powder of high purity, we did not consider any preferred orientation/texture effects in our refinements.

## 3. Results and discussion   

All observed diffraction patterns collected at different times and temperatures during hydrogenation and dehydrogenation are shown in Fig. 3 ▸ as a two-dimensional plot, viewed down the intensity axis, with diffraction pattern number, time and temperature along the ordinate, and peak positions along the abscissa. The corresponding heat treatment history is shown on the side of each measurement with the same time scale. All observed phases are colour coded in the thermal history curve and their corresponding labels shown on top of the figure. The temperature ranges of all these phases are also noted with numbers in the diffraction map.

**Figure 4:**
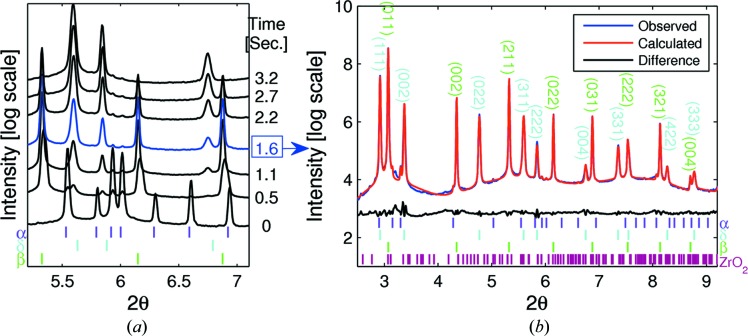
(*a*) Diffraction patterns from region 2 in Fig. 3 ▸. (*b*) The Pawley refinement result of the marked (blue) diffraction pattern in (*a*).

For clarity, and to show the rate of the phase transformation together with the evolution of existing phases, the phase transition region number 2 marked in Fig. 3 ▸ is shown as a waterfall plot in Fig. 4 ▸(*a*). The relative acquisition time with respect to the first diffraction pattern is also shown on the left side of each pattern with the same colour code. The *HKL* peak positions of various phases in Fig. 4 ▸, and in all other figures in the paper, are indicated with colour-coded tick marks. The corresponding phase names of those tick marks are also noted with the same coloured text adjacent. In order to demonstrate the quality of the data, a Pawley refinement result of the diffraction pattern collected for 1.6 s (relative time) marked with the blue arrow in Fig. 4 ▸(*a*) is shown in Fig. 4 ▸(*b*).

Despite careful powder handling, visible traces of Zr oxide were observed in some patterns and they can be seen in both Fig. 3 ▸ and Fig. 4 ▸. To avoid confusion, their indices are only shown in Fig. 4(*b*) here.

**Figure 5:**
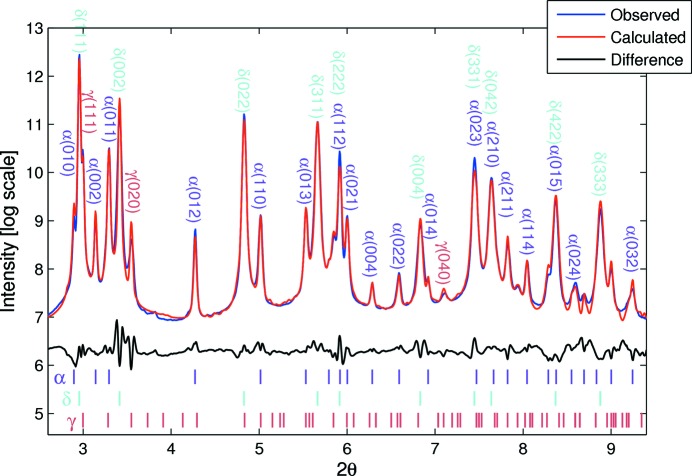
Rietveld refinement result of last measurement (black) in region 7 in Fig. 3 ▸. The profile in red is the calculated pattern.

From the diffraction map in Figs. 3 ▸ and 4 ▸, one can clearly see that at the beginning of charging there is only pure α-Zr phase in the system. After introducing hydrogen gas at 313°C, the α-Zr phase quickly transforms into the δ-phase + β-Zr phase. Then, the β-Zr phase promptly transforms into the δ-ZrH_*x*_ phase region. Moving on, δ-ZrH_*x*_ gradually transforms into hydrogen-rich f.c.t. ∊-ZrH_*x*_ phase. This whole transition from the α-Zr phase to ∊-hydride phases was completed in less than 30 s. After completion of the δ to ∊ transition, there were no structural changes observed for about an hour. In order to see the reversibility of this transition, we raised the temperature to 613°C and degassed the system. As we raised the temperature, the hydrogen-rich ∊-ZrH_*x*_ phase can be observed to continuously transform back to stoichiometric, cubic δ hydride at a temperature of about 518°C. During the temperature increase, a fraction of the δ-ZrH_*x*_ phase transformed into α-Zr phase at 556°C. After reaching 613°C, we started to decrease the temperature and observed that the amount of α-Zr phase increased slightly and the controversial γ-ZrH_*x*_ was observed at 180°C. However, at room temperature the amount of δ-hydride phase which transformed into α-Zr and γ-hydride phases was not substantial. At the end of the measurement we still had 81 wt% of δ-hydride in our system (Fig. 5 ▸). As most of the γ-ZrH peaks are located in very close proximity of δ-hydride and α-Zr matrix peaks and their relative intensities are not very strong, only three clearly visibly peaks are indexed in Fig. 5 ▸. Based on measured temperatures and existing phases in each collected diffraction pattern, approximate phase diagram locations of observed phases noted by 1 to 7 in Fig. 3 ▸ are shown with blue numbered points in Fig. 1 ▸.

To our knowledge, the phase transformation between all the phases in the Zr–H system has not been recorded *in situ*. Thanks to high-energy synchrotron X-rays, the large two-dimensional detector, and the flexible high-temperature and high-pressure gas loading system at ESRF, we were able to capture all these reported low-pressure phases in one single run. From Fig. 3 ▸, it is also evident that with this setup the kinetic behaviour of phase transformations can be observed in real time even though, in one case, measurement was completed in less than 0.2 s (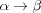 transformation).

According to Figs. 3 ▸ and 4 ▸, the β-Zr phase was observed at 313°C together with δ-ZrH_*x*_. However, from the Zr–H phase diagram (Fig. 1 ▸) it is clear that the β-Zr + δ-ZrH_*x*_ phase only co-exists above 550°C, depending on hydrogen concentration. From the Rietveld analysis, we found that there is a significant difference between published lattice parameters for the β-Zr phase and what we found in our studies. The lattice parameter of the β-Zr phase, which we observed, is 4% larger than the reported lattice parameter of the β-Zr phase at 863°C (Zuzek *et al.*, 1990 ▸). In addition, the calculated lattice parameters of δ-hydride that formed after the β to δ transformation are marginally larger than the lattice parameters obtained by Singh *et al.* (2007 ▸) at 500°C. These indicate that the actual temperature of the Zr powder in the reaction cell when the β-Zr phase was observed was significantly higher than that measured by the thermocouple connected to the sample holder for a couple of fractions of a second during the early stages of the reaction. We believe that such a high temperature rise occurs because of the very high reactivity of a powder (compared with a solid) caused by its higher surface-to-bulk ratio. The heat produced during the exothermic reactions of hydrogen solvation and hydride formation would, thus, raise the local temperature of the powder significantly for a brief time and not be recorded by the thermocouple situated some distance away from the powder on the sample holder. In order to avoid such temperature mismatch, we suggest (1) to use an extra thermocouple or place the existing thermocouple closer to the reactant powder; (2) mix Zr powder with another well defined standard powder sample to use them as a reference for extracting the temperature from the thermal expansion; (3) reduce the hydrogen loading pressure to slow down the hydrogen absorption rate.

The metastable trigonal ζ-Zr hydride which belongs to space group *P*3*m*1 reported by Zhao *et al.* (2008 ▸) was not observed in the whole of the hydrogenation and dehydrogenation process. The possible reasons are: (1) the presence of different impurity or alloying elements; (2) the formation and transformation rate of this phase might be much faster than the data acquisition rate. In order to define the existence and transformation mechanism of the ζ phase, more experiments are needed with shorter data acquisition times and also different Zr and Zr alloy samples.

As shown in Fig. 5 ▸, the similarity of both peak intensities and their variations, as well as background intensities, is good, which gives us confidence that the measurement and analysis are good. The lattice parameters of the α-Zr phase after post-heat treatment obtained in this study (*a* = 3.24205 Å and *c* = 5.16645 Å) are in very good agreement with those given by Zuzek *et al.* (1990 ▸). During heat treatment, the α-Zr phase showed close to linear thermal expansion behaviour with respect to temperature. After cooling from 613°C to room temperature, we did not observe exactly the same lattice parameters for the α-Zr phase; instead we found a slightly expanded structure, indicated with an asterisk in Table 1 ▸. The small expansion of the unit cell of the α-Zr phase at room temperature after annealing is likely to be the effect of small amounts of residual hydrogen still left in the matrix phase. The lattice parameters of δ- and γ-ZrH_*x*_ after heat treatment are in good agreement with data published in the literature.

## 4. Conclusions   

To identify the formation mechanisms, stability and crystal structure of various Zr hydrides, for the first time an *in situ* hydrogenation study was performed on high-purity Zr powder at a third-generation synchrotron X-ray radiation facility, and all commonly reported low-pressure phases presented in the Zr–H phase diagram are obtained in a single setup.

The present study not only demonstrates the potential use of high-energy synchrotron diffraction in the *in situ* measurement of phase transformation and identification of phases in the Zr–H system, but also shows the possibility of observing the kinetic behaviour of phase transformations in real time even as measurement, in one case, was completed in less than 0.2 s in the case of α → β.

Despite the slow cooling rate, the elusive γ-ZrH_*x*_ phase was observed at 180°C, and the complete reversible transformation between ∊ and δ was recorded. The hydrogen-rich ∊-phase reversibly transforms first to pure δ-phase and then α-Zr + δ-hydride as a function of H/Zr ratio as hydrogen was degassed from the system at 600°C.

In order to obtain precise temperature measurements, it is suggested that an extra thermocouple is used or the existing one is placed as close as possible to the reactant. In addition, using reference powder is advised.

## Figures and Tables

**Table 1:** Some known and calculated crystallographic information for Zr and Zr hydrides

Phase	Structure	Space group	*a* (Å)	*b* (Å)	*c* (Å)	Temperature (°C)	Reference
α(Zr)	h.c.p.	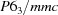	3.2316	3.2316	5.1475	25	Zuzek *et al.* (1990 ▸)
			3.24205	3.24205	5.16645	25	Current studies
			3.25721	3.25721	5.19881	25*	
β(Zr)	b.c.c.	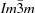	3.6090	3.6090	3.6090	863	Zuzek *et al.* (1990 ▸)
			3.75788	3.75788	3.75788	300	Current studies
δ(ZrH_1.66_)	f.c.c.	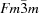	4.7783	4.7803	4.7803	20	Zuzek *et al.* (1990 ▸)
			4.8051	4.8051	4.8051	500	Singh *et al.* (2007 ▸)
			4.84159	4.84159	4.84159	300	Current studies
			4.77854	4.77854	4.77854	25*	
∊(ZrH_2_)	f.c.t.	*I*4/*mmm*	4.9689	4.4689	4.4497	20	Zuzek *et al.* (1990 ▸)
			4.9743	4.9743	4.52469	300	Current studies
γ(ZrH)	f.c.t.	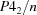	4.592	4.592	4.970	17	Zuzek *et al.* (1990 ▸)
			4.59966	4.59966	4.98654	25*	Current studies
ζ(Zr_4_H)	Trigonal	*P*3*m*1	3.3	3.3	10.29	–	Zhao *et al.* (2008 ▸)

* Result at 25°C after sample had been annealed.

**Table 2:** Nominal composition of the Zr-powder specimens studied in this study

wt%	Impurity elements (p.p.m.)						
Zr	C	Hf	Fe	Cr	N	O	H
99.2	250	2500	200	200	100	1000	10
